# The Week's Work

**Published:** 1911-04-22

**Authors:** 


### The Week's Work.

### THE FLORENCE NIGHTINGALE MEMORIAL.

Miss Mollett, the matron of the Royal South Hants and Southampton Hospital, has rendered a service to all those who are interested in the work and efforts of the late Florence Nightingale by drawing attention, in the columns of the lay press, to the manner in which the Executive Committee of the Nightingale Memorial Fund has dealt with the matter of selecting the form which the memorial is to take. The General Committee, of which Miss Mollett is a member, was not called together, and has never had an opportunity of discussing the proposals put forward by the Executive Committee —omissions which are almost unthinkable when it is borne in mind that these proposals interest the whole nursing and hospital world. It is unheard of that the decision as to the ultimate form of the memorial should be allowed to rest in the hands of a few persons who, judging from the correspondence which has followed Miss Mollett's letter, are evidently not in touch with public feeling on the subject. It is equally absurd to imagine that the public will contribute towards a fund controlled by a committee which exists, apparently, only in name, and which, it seems, has not had an opportunity of conferring together and expressing its approval or otherwise of the proposals put before the general meeting at the Mansion House. In these circumstances it is of the utmost importance that the General Committee should be convened as soon as possible to deliberate on the proposals submitted and the manner in which they are to be carried out. This course is imperative if we wish to preserve that unanimity and enthusiasm without which the memorial scheme cannot but result in a failure as lamentable as it would be inexcusable.

### A REQUEST TO OUR READERS.

Institutional secretaries are beginning generally to realise the importance of having the histories of their institutions written, and, that a record of all those already published may be available, the Editor asks to hear from every hospital where this has been done. Whether the history has been written in two large volumes or summarised in an authoritative monograph, information concerning it will be equally welcome. The title, the price, and the publisher's name should be sent in each case. A really important service can be rendered to the hospital world by a little co-operation on the part of our readers, whom we thank in advance, believing that they will promptly realise its value.

### "THE HOSPITAL AND THE YOUNG PHYSICIAN."

Under this title Dr. Edgar Darnall contributes an interesting paper to the current number of the *New York Medical Record.* "Since the rapid development of hospitals," he remarks, "there have been added to the usual difficulties of keen competition, high cost of living, youth, inexperience, lack of adequate acquaintance, and the other drawbacks the young private practitioner has to face, the serious competition of hospitals and dispensaries. . . . He waits for work while the hospital eats up his bread and butter." The author proceeds to deal in detail with the various items in the indictment he draws up against what may reasonably be termed thoughtless institutional competition. Who nowadays thinks of calling in a neighbouring doctor to a street accident? The ambulance is promptly called for and the patient taken to the nearest hospital. The young doctor is forced to accept contract practice in very self-defence.

### THE OTHER SIDE OF THE SHIELD.

This is the one side of the shield. Dr. Darnall admits that there is another side to it on which the colours are much brighter and the picture much more pleasant to look upon. "To the young man even of good ability but without financial backing or influence the competition of hospitals may seem in the beginning the greatest barrier to success, yet if he is fortunate enough to get himself connected with some hospital in a capacity however humble at first, the hospital may be of the greatest service to him. Its advantages professionally so far outweigh the disadvantages financially that they are not to be compared. It is undoubtedly true that there are many good men who are forced out into private practice improperly equipped because, although endowed with the necessary ability to make good clinicians, the financial necessity of gaining an immediate income is such that they are deterred from taking dispensary positions, which would give them experience and skill, because they cannot spare the large amount of time required and for which they get no money remuneration." The greatest good the hospital does in any community is not so much what it does for the individual as the good it does in sending out men trained to the scientific habit of mind, and whose influence and authority as leaders are reflected in the general diffusion of sanitary knowledge in the community. The value of a hospital to a community is directly expressed by the individual value of the trained minds of its staff to the community. The influence of such trained minds must necessarily elevate the general professional average by setting a high standard, to which others must at least try to approximate.

### A PLEA FOR PAYMENT OF HOSPITAL STAFFS.

The article, which is a thoughtful and readable exposition of the case for and against the general practitioner in relation to hospitals, is a sturdy plea on behalf of the principle that all workers should be paid. By offering adequate remuneration to the resident staff an institution will be able to secure good men who are under present conditions prevented from serving as house physicians or surgeons owing to the fact that they have no means to defray the expenses which such a period of free labour involves. At present it is the well-to-do student alone who can afford to take a house appointment at a large London hospital. He gets no pay for his work. If he is a house surgeon he may make a fair wage by giving anæsthetics or dressing to his principal in private cases, but he has to pay for the six months' added experience that he gets. The result has been that the good men who are not blessed with a banking account go to provincial hospitals, where they are paid and where the experience is as good as, if not better than, what can be gained at a London hospital with an attached teaching school. The chain of results which follow such a policy is easy to sketch. The institution itself loses, the profession loses—owing to the fact that inferior men, who through their financial independence have been able to hang on, ultimately become members of the teaching staffs—and the public loses. All this Dr. Darnall admits heartily, and his conclusion, though not expressly stated, is too obvious to be overlooked. The resident staff must be paid, and adequately, if a hospital is to obtain the best from its medical school and from those who have been taught in its wards.

### THE WASTE OF ANÆSTHETICS.

We have lately been interested, in tabulating the expenditure on anæsthetics at several hospitals, to find that there is a marked and, in our opinion, an unwarranted increase in the bill for anæsthetics at institutions which have favoured the open ether method to the exclusion of other methods of administration. This method is admittedly wasteful, especially in the hands of a student or tyro or where the patient is operated upon without previously receiving an injection of some narcotic or anodyne. Captain Carruthers, R.A.M.C., recently mentioned the fact that he had seen "sixteen ounces of ether used for an ordinary appendicitis operation," and we have ourselves witnessed a similar waste on more than one occasion where the open method is used. There is, so far as we are aware, no special reasons why this method should be the routine in hospital practice. It is not a more safe method than the inhalation of ether by other and less wasteful methods, and however admirably adapted to the needs of the private practitioner, it is not the method of choice to be used in institutions. The anæsthetic bill, we imagine, can be much reduced by the use of apparatus such as the Roth Draeger or the Vernon Harcourt, both of which are so economical that they soon repay their initial cost through the saving in anæsthetics which their use effects. This is a matter to which hospital secretaries might with advantage pay some attention. No doubt the comparative increase in the anæsthetic bill is accounted for by the increase in the number of operations and the slight increase in the cost of ethylic ether, but this cannot account for such increased expenditure in every case. Where ethylic ether is used the bill is, of course, much higher than where the cheaper methylic ether is employed. That, however, is a matter which need not concern us; though we are of opinion that methylic ether is in every way as safe and as reliable as the higher-priced drug, and that wherever possible the former should be supplied unless the anæsthetist specially asks for ethylic ether.

### THIS WEEK'S DRUG MARKET.

In consequence of the Easter holidays, business has been suspended for the greater part of the week and will probably not be resumed in earnest until the beginning of next week. Opium is dearer, as also are morphine and codeine. Cod-liver oil tends lower in price. An advance in the price of potassium bromide and other bromide salts is thought to be imminent.

